# Ascending Aorta Diameter Changes after Aortic Valve Replacement in Elderly Patients with Aortic Valve Stenosis

**DOI:** 10.1155/2022/5509364

**Published:** 2022-09-10

**Authors:** Jiamiao Gong, Kang An, Hongyuan Lin, Jianfeng Hou

**Affiliations:** Adult Cardiac Surgery Center, National Center for Cardiovascular Diseases and Fuwai Hospital, CAMS and PUMC, Beijing 100037, China

## Abstract

**Objective:**

To describe the natural history of the ascending aorta in elderly patients after aortic valve replacement (AVR) for aortic valve stenosis and to clarify the risk factors associated with the progression of the ascending aorta.

**Methods:**

This retrospective review included a total of 87 elderly patients who had undergone aortic valve replacement for severe aortic valve stenosis in Fuwai Hospital. The patients were categorized into two groups based on the height-based aortic height index (AHI) before AVR, as determined by echocardiography and computed tomography: Group *A* (*n* = 28) was defined as an AHI > 2.44 cm/m, and Group *B* (*n* = 59) was defined as an AHI ≤ 2.44 cm/m. The perioperative and follow-up data were collected, and a linear mixed-effect model was used to analyze and compare the change rate of the ascending aorta after AVR.

**Results:**

The mean follow-up period was 4.0 ± 1.3 years. The diameter of ascending aorta in group *A* increased from 37.2 ± 5.0 mm at discharge to 40.7 ± 4.7 mm at the last follow-up (*P*=0.001), while that of group *B* increased only from 33.3 ± 4.4 mm to 33.7 ± 4.1 mm (*P* > 0.05).The ascending aorta diameter expansive rate was 0.81 mm/year in group *A* and 0.14 mm/year in group *B*. The expansive rate was significantly greater in patients with an AHI>2.44 cm/m than in those with anything else (*P* = 0.009). A univariable linear mixed model analysis revealed that the AHI>2.44 cm/m was the only significant risk factor for ascending aortic dilatation rate after AVR. There were 4 patients who died in hospital and 11 late follow-up deaths. Particularly, there was no aortic event that occurred during follow-up.

**Conclusion:**

For elderly patients with aortic stenosis, the possibility of progressive ascending aortic dilatation after AVR demands regular follow-up, and AHI may be an important risk factor for the change rate of the diameter of the ascending aorta.

## 1. Introduction

Because of the progress of population aging, the proportion of aortic valve disease in elderly patients is increasing gradually. The hemodynamic changes caused by severe aortic stenosis, the variation of the aortic wall associated with advanced age and also the genetic factors such as bicuspid aortic valve, accelerate aortic dilatation [1–4]. Aortic valve replacement (AVR) has been proven to be an effective surgical treatment, but it was still reported that the expansion of the ascending aorta continued [5–7]. Preventative concomitant ascending aortic replacement may be a solution, but it increases mortality and complications, especially in the elderly population [8]. As transcatheter aortic valve replacement (TAVR) has recently become widely accepted in the elderly population, it is also crucial to consider the indications. Therefore, it is necessary to elucidate the natural history of ascending aortic dilatation and to evaluate the indications of ascending aortic surgery.

In international guidelines, surgical intervention criteria for ascending aortic aneurysm are based on an absolute raw aortic diameter ≥55 mm and between 45 and 50 mm for various particular or genetic aortopathies, such as bicuspid and Marfan syndrome [9, 10]. A main weakness of using absolute aortic diameter is the inability to factor in a significant determinant of aortic dimensions: the patient's body size. This may underestimate the risk in some small-stature patients. Furthermore, indexing patient height to aortic dimensions has been shown to enhance mortality prognostication in patients with TAAA [11].

Therefore, the purpose of this retrospective study was to elucidate the natural history of the ascending aorta following AVR in elderly patients and to use a simple height-based relative aortic size measure, the aortic height index (AHI), defined as aortic size divided by patient height, to identify risk factors for progressive aortic dilatation.

## 2. Materials and Methods

### 2.1. Study Design and Patients

**Figure 1:**
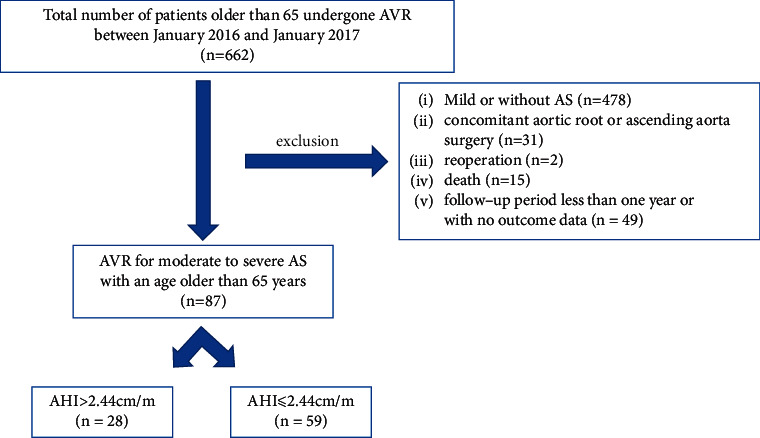
Patient flow diagram used in the study.

All patients provided written informed consent for the anonymous use of their data, and this retrospective clinical study received approval from the Institutional Review Board at Fuwai Hospital (approval number 2021-1477). Between January 2016 and January 2017, we performed AVR on 662 patients who were older than 65 years at our institution. And diagnosed with moderate to severe aortic valve stenosis at our institution. Patients were excluded who had undergone concomitant aortic root or ascending aorta surgery (*n* = 31), or with less than moderate AS (*n* = 478), reoperation (*n* = 2), death (*n* = 15), and a follow-up period of less than one year or with no outcome data (*n* = 49). This left us with 87 patients who were finally included in this retrospective review. We categorized the enrolled patients into two groups based on the height-based aortic height index (AHI), which was defined as aortic diameter/height (cm/m). Group *A* (*n* = 28) was defined as those patients with an AHI > 2.44 cm/m, while Group *B* (*n* = 59) was defined as those patients with an AHI ≤ 2.44 cm/m (Figure 1).

### 2.2. Imaging

All patients underwent transthoracic echocardiography (TTE) and enhanced computed tomography (CT) one week before the operation. We also performed TTE regularly starting three months after the operation. All echocardiography was performed by professional senior doctors. According to the recommendations of the American Society of Echocardiography and the European Society of Cardiovascular Imaging [12, 13]. We measured the diameter of the ascending aorta at the tubular portion in the end-diastole from the leading edge of the anterior aortic wall to the leading edge of the posterior aortic wall, on the parasternal long-axis view, perpendicular to the long axis of the aorta. We performed the scan, measuring the diameter of the ascending aorta at the tubular portion, taken in diastole, and the distance from intima to intima was measured. Referring to the previous research, the height-based aortic height index (AHI) may be an effective method to indicate the risk of aortic complications by eliminating the influence of body shape, and the best cut-off value for moderate risk of complications is 2.44 cm/m [14]. Therefore, we categorized the enrolled patients into two groups based on AHI, with a dividing line of 2.44 cm/m.

### 2.3. Follow-up

The follow-up was mainly completed through consultation and telephone. The follow-up examination was completed at 3 months, 6 months, and every year after the operation. Echocardiography was regularly performed during follow-up, and if the ascending aorta was significantly dilated, CT was settled. The main adverse cardiovascular events and aortic events were recorded.

### 2.4. Statistical Analyses

All statistical analyses were performed using SPSS 26 (SPSS, Inc., Chicago, IL, USA). Continuous variables were expressed as the mean ± standard deviations, and categorical variables are expressed as frequency and percentage. The two groups were compared using the chi-squared test or Fisher's exact test for categorical variables and an unpaired Student's *t*-test for continuous variables. The linear mixed-effects model was applied to compare the ascending aorta diameter changes after AVR between the two groups. In the mixed-effects model, *P* values that were obtained by analyzing changes in the aortic diameter in relation to time were considered as covariates × time interactions. Time was measured in years. The slopes of changes in the aortic diameter were calculated. As for preoperative variables, we analyzed sex, hypertension, more than moderate aortic regurgitation, presence of bicuspid aortic valve (BAV), preoperative AHI, and type of the prosthetic valves (bioprosthetic or mechanical). *P* values <0.05 were considered statistically significant for all analyses.

## 3. Results

### 3.1. Baseline Characteristics

Table 1 shows the preoperative patient characteristics. Finally, 87 survival patients who completed at least 1 year follow-up examination were included. Two groups were divided based on AHI. Group *A* (*n* = 28) was defined as AHI > 2.44 cm/m, while Group *B* (*n* = 59) was defined as AHI ≤ 2.44 cm/m. In group *A*, the majority of the ascending aorta size (82.1%) was between 40 and 50 mm, only 4 patients (14.3%) were less than 40 mm. On the contrary, in group *B*, the majority of the ascending aorta size (93.2%) was less than 40 mm, only 4 patients (6.8%) had more than 40 mm. The baseline data of the two groups were similar. There was no difference associated with aortic valve disease, history, and perioperative data, except for the diameter of the left ventricle.

### 3.2. Ascending Aorta Diameter Changes after Aortic Valve Replacement

The mean follow-up was 4.0 ± 1.3 years in group A and 4.1 ± 1.3 years in group B. The diameter of ascending aorta in group A increased from 37.2 ± 5.0 mm at discharge to 40.7 ± 4.7 mm at the last follow-up (*P*=0.001), while that of group B increased only from 33.3 ± 4.4 mm to 33.7 ± 4.1 mm (*P* > 0.05) (Table 2).

**Figure 2:**
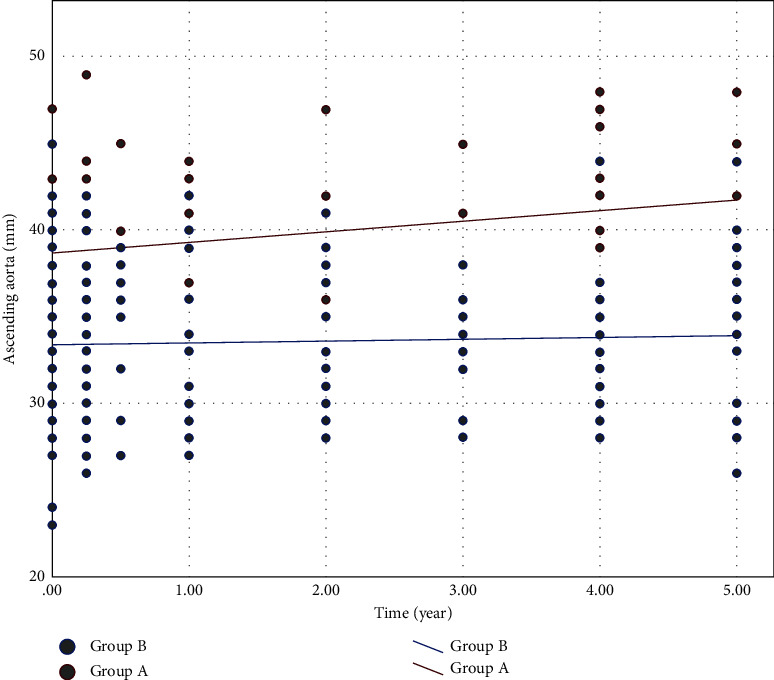
Dot plot depicting changes in the ascending aorta over five years after AVR in individual patients in group *A* (red dots) and group *B* (blue dots). Red line indicates the overall enlargement rate of the ascending aorta in group *A* calculated according to a linear mixed model. Blue line indicates the overall enlargement rate of the ascending aorta in group *B* calculated according to a linear mixed model.

We compared the slopes of ascending aortic change between the two groups. The ascending aorta diameter expansive rate was 0.81 mm/year in group *A* and 0.14 mm/year in group *B*. The enlargement rate was significantly higher in patients with AHI > 2.44 cm/m than those in else (*P*=0.009) (Figure 2). A univariable linear mixed model analysis revealed that the AHI > 2.44 cm/m was the only significant risk factor for ascending aortic dilatation after AVR (Table 3).

Among the included patients, there was only one complication of secondary thoracotomy for hemostasis on the first day after the operation. There were 4 patients who died in hospital after the operation, 11 died during the follow-up period, and 10 failed to be followed up. Among the 4 patients who died in hospital, one died of renal failure and multiple organ failure, one for prosthesis-patient mismatch and heart failure, one for coronary disease and heart failure, and the last caused by liver failure. Only one patient (25%) had an AHI more than 2.44 cm/m, with an ascending aorta size of 49 mm, who died from liver failure. There were 11 late deaths during the follow-up period. The causes of death were heart failure in 2 patients, myocardial infarction in 1 patient, cerebrovascular accident in 2 patients, malignant tumor in 4 patients, and 2 dead for unknown reasons. Three of them (27.3%) had an AHI more than 2.44 cm/m, with an ascending aorta size of 45, 40, and 44 mm, who died from cerebrovascular accident, gastric cancer, and unknown reasons. Particularly, there was no aortic event that occurred during follow-up.

## 4. Discussion

Among healthy people, the diameter of the aorta was mainly related to age, gender, body shape, and blood pressure [15, 16]. The elastic fibers of the aortic wall decreased significantly with age, while the composition of collagen fibers increased, which increased the vulnerability and ease of remodeling of the aortic wall in elderly patients [4]. In our study, we found that only 32.9% of patients could avoid the expansion of the ascending aorta after AVR. Tsutsumi et al. described the natural history of ascending aorta diameter after AVR and showed that only 17.4% of patients did not have ascending aortic dilatation during follow-up [17]. This indicated that the increase in the ascending aorta diameter was universal, and AVR could not completely avoid the risk of ascending aortic dilation.

In our study, the preoperative ascending aorta diameter is the only risk factor for the change rate of the ascending aorta after operation. According to Laplace's law, the aortic wall stress was directly proportional to the aortic diameter. Therefore, the dilated ascending aorta brought greater wall pressure. Meanwhile, natural histological changes of aortic wall tissue in elderly patients and the blood flow impact caused by aortic valve disease accelerated the expansion process [4, 18]. In international guidelines, surgical intervention criteria for the ascending aorta were based on absolute raw aortic diameter. However, these criteria for risk estimation ignored a significant factor determinant of aortic dimension: the patient's body size [19]. Moreover, weight fluctuates during a lifetime and can be deliberately influenced. Based on the study by Zafar et al., an AHI of 2.44 to 3.17 cm/m indicated a moderate risk of aortic complications, and an AHI of 3.21 to 4.06 cm/m was at high risk [14]. According to our study, in group *A*, the majority of the ascending aorta size (82.1%) was between 40 and 50 mm. By contrast, in group *B*, the majority of the ascending aorta size (93.2%) was less than 40 mm. This was basically matched with the absolute aorta size classification. Therefore, the height-based aortic height index (AHI) could be simple but effective method to evaluate and categorize the risk.

In the present study, although the aortic diameter showed a progressive dilation in patients with a higher AHI, no aortic events were found during the whole follow-up period. In other words, the ascending aorta remained stable during follow-up even though it existed with a different expansion rate. Previous articles also showed that the incidence of aortic events after AVR was low. Girdauskas et al. reported that fifteen-year freedom from adverse aortic events was 93 ± 3% in the bicuspid aortic valve group versus 82 ± 6% in the tricuspid aortic valve group [20]. However, in the elderly population, this should be cautious because of the histological and hemodynamic differences. Reoperation in elderly patients could be a real knock to the prognosis and significantly increase the mortality and risk of complications. Based on the results of this study, an AHI of more than 2.44 cm/m warranted at least regular radiographic follow-up to monitor the expansion trend due to the fact of progressive dilation.

Our study indicated that the bicuspid aortic valve did not affect the changes in the ascending aorta after operation. According to the current American Thoracic Surgery Association guidelines, the surgical intervention criteria for ascending aortic in BAV is greater than 45 mm. While there is still controversy about the slightly dilated ascending aorta of 40–45 mm. Longi et al. demonstrated that in bicuspid patients with mild dilation of the ascending aorta (40–45 mm), proximal aortic dilation is a very slow process, which is not significantly different from those in the group less than 40 mm [21]. Another research by Girdauskas et al. showed a stable diameter of ascending aorta after AVR in BAV group with preoperative ascending aorta ≥40 mm, which had an expansion rate as low as 0.09 mm/year·patient, even the same in those of the ascending aorta ≥50 mm [22]. Based on the low incidence of aortic events previously reported, it is not recommended for aggressive surgical intervention of the ascending aorta.

### 4.1. Limitation

We acknowledge that there are several limitations to this study. Firstly, this was a retrospective study, and secondly, the study population was relatively small in number. Thirdly, the follow-up measurement of the ascending aorta was performed by echocardiography and CT imaging, but not all by CT imaging, although transthoracic echocardiography is proven to be an available and accurate technique. Besides, the preoperative data on ascending aorta, there was not much difference between echocardiography and CT scan. It was therefore considered acceptable to record echocardiographic data in the follow-up examination, in part because of the radiation exposure.

## 5. Conclusion

For elderly patients with aortic stenosis, the possibility of progressive ascending aortic dilatation after AVR demands regular postoperative evaluation and follow-up, and AHI may be an important risk factor for the change rate of the diameter of ascending aortic.

## Figures and Tables

**Table 1:** Patient characteristics.

	Total (*n* = 87)	Group *A* (*n* = 28)	Group *B* (*n* = 59)	*P*
Age (year)	69.9 ± 3.4	69.7 ± 3.0	69.9 ± 3.6	0.75
Sex (female)	39 (44.8%)	17 (60.7%)	22 (37.3%)	0.04
Height (cm)	163.2 ± 8.3	161.1 ± 9.5	164.0 ± 7.6	0.13
Weight (kg)	63.5 ± 11.1	61.5 ± 12.0	64.5 ± 10.6	0.24
BSA (m^2^)	1.66 ± 0.2	1.62 ± 0.2	1.67 ± 0.2	0.21
Hypertension	40 (46%)	9 (32.1%)	31 (52.5%)	0.08
Diabetes	10 (11.5%)	1 (3.6%)	9 (15.3%)	0.11
Smoke	54 (62%)	27 (96.4%)	57 (96.6%)	0.96
Hyperlipidemia	39 (44.8%)	14 (50%)	25 (42.4%)	0.51
COPD	1 (2.2%)	0	1 (1.7%)	0.02
Cerebrovascular disease	1 (1.1%)	1 (3.6%)	0	0.15
Atrial fibrillation	8 (9.2%)	3 (10.7%)	5 (8.5%)	0.74
Coronary artery disease	24 (27.6%)	6 (21.4%)	18 (30.5%)	0.37
NYHA (≧3)	43 (49.4%)	13 (46.5%)	30 (50.9%)	0.67
Moderate and severe aortic regurgitation	32 (36.8%)	12 (42.9%)	20 (33.9%)	0.42
Presence of BAV	39 (44.8%)	15 (53.6%)	24 (40.7%)	0.26
Echocardiography				
Ejection fraction (%)	60.1 ± 8.3	58.9 ± 8.0	60.7 ± 8.4	0.34
Left ventricular diameter (mm)	51.1 ± 8.1	54,6 ± 8.7	49.5 ± 7.2	0.006
Peak velocity (m/s)	4.7 ± 0.8	4.8 ± 0.8	4.6 ± 0.8	0.44
Mean gradient (mmHg)	55.9 ± 19.6	57.8 ± 20.1	54.9 ± 19.5	0.54
Ascending aorta (mm)	37.0 ± 5.9	43.4 ± 3.8	34.0 ± 3.9	<0.01
<40	59 (67.8%)	4 (14.3%)	55 (93.2%)	
40–50	27 (31.1%)	23 (82.1%)	4 (6.8%)	
>50	1 (1.1%)	1 (3.6%)	0	
AHI (cm/m)	2.27 ± 0.37	2.69 ± 0.21	2.07 ± 0.23	<0.01
Prosthetic valve				
Bioprosthetic	68 (78.2%)	22 (78.6%)	46 (78%)	0.95
Mechanical	19 (21.8%)	6 (21.4%)	13 (22%)	0.95
Prosthetic valve size (mm)	21.7 ± 1.8	21.8 ± 1.6	21.6 ± 1.9	0.76
Concomitant procedure				
CAGB	24 (27.6%)	6 (21.4%)	18 (30.5%)	0.37
Mitral valve	15 (17.2%)	6 (21.4%)	9 (15.3%)	0.48
Tricuspid valve	13 (14.9%)	6 (21.4%)	7 (11.9%)	0.29
Maze	1 (1.1%)	0	1 (1.7%)	0.49
Cardiopulmonary bypass time (min)	117.0 ± 50.4	118.1 ± 62.3	116.5 ± 44.5	0.90
Clamp time (min)	90.2 ± 40.7	87.4 ± 44.1	91.4 ± 39.4	0.67
Follow-up (year)	4.0 ± 1.3	4.0 ± 1.3	4.1 ± 1.3	0.72

BSA, body surface area; COPD, chronic obstructive pulmonary disease; NYHA, New York heart association. BAV, bicuspid aortic valve; AHI, aortic height index; and CABG, coronary artery bypass grafting.

**Table 2:** Postoperative data.

	Total (*n* = 87)			Group *A* (*n* = 28)			Group *B* (*n* = 59)		
	Discharge	Last follow-up	*P*	Discharge	Last follow-up	*P*	Discharge	Last follow-up	*P*
Ejection fraction (%)	60.1 ± 7.2	62.1 ± 7.1	0.046	59.2 ± 7.8	61.6 ± 6.1	0.22	60.6 ± 7.0	62.3 ± 7.6	0.12
Left ventricular diameter (mm)	45.3 ± 5.3	46.1 ± 4.8	0.19	45.4 ± 4.8	46.8 ± 5.0	0.15	45.3 ± 5.5	45.8 ± 4.8	0.50
Peak velocity (m/s)	2.5 ± 0.5	2.6 ± 0.7	0.046	2.4 ± 0.4	2.7 ± 0.7	0.064	2.5 ± 0.5	2.6 ± 0.7	0.28
Ascending aorta (mm)	34.7 ± 4.9	36.0 ± 5.4	0.012	37.2 ± 5.0	40.7 ± 4.7	0.001	33.3 ± 4.4	33.7 ± 4.1	0.56

**Table 3:** Ascending aorta diameter changes after AVR using univariable linear mixed model.

Variables	*B* ± standard error	*P*
Sex (female)	−0.22 ± 0.24	0.35
Hypertension	0.17 ± 0.23	0.45
Moderate and severe aortic regurgitation	−0.30 ± 0.24	0.22
Presence of BAV	0.073 ± 0.24	0.76
Prosthetic valve (bioprosthetic)	0.29 ± 0.28	0.30
AHI > 2.44	0.68 ± 0.25	0.009

## Data Availability

All relevant data are included within the manuscript, and any other additional data are available upon request from the corresponding author.
